# VARSCOT: variant-aware detection and scoring enables sensitive and personalized off-target detection for CRISPR-Cas9

**DOI:** 10.1186/s12896-019-0535-5

**Published:** 2019-06-27

**Authors:** Laurence O. W. Wilson, Sara Hetzel, Christopher Pockrandt, Knut Reinert, Denis C. Bauer

**Affiliations:** 1 CSIRO, Sydney, NSW Australia; 2 Freie Universität, Berlin, Germany; 3 Max Planck Institute for Molecular Genetics, Berlin, Germany

**Keywords:** CRISPR-Cas9, Off-target detection, Variants, Genome editing

## Abstract

**Background:**

Natural variations in a genome can drastically alter the CRISPR-Cas9 off-target landscape by creating or removing sites. Despite the resulting potential side-effects from such unaccounted for sites, current off-target detection pipelines are not equipped to include variant information. To address this, we developed VARiant-aware detection and SCoring of Off-Targets (VARSCOT).

**Results:**

VARSCOT identifies only 0.6% of off-targets to be common between 4 individual genomes and the reference, with an average of 82% of off-targets unique to an individual. VARSCOT is the most sensitive detection method for off-targets, finding 40 to 70% more experimentally verified off-targets compared to other popular software tools and its machine learning model allows for CRISPR-Cas9 concentration aware off-target activity scoring.

**Conclusions:**

VARSCOT allows researchers to take genomic variation into account when designing individual or population-wide targeting strategies. VARSCOT is available from https://github.com/BauerLab/VARSCOT.

**Electronic supplementary material:**

The online version of this article (10.1186/s12896-019-0535-5) contains supplementary material, which is available to authorized users.

## Background

The development of the CRISPR-Cas9 system has revolutionized genome-editing [1]. The system can be targeted to almost any genetic sequence through complementary binding to an associated gRNA. Once cleaved, the repair of the break can be manipulated to induce small insertions or deletions or used for the insertion of new sequence [2, 3]. This has significant implications, particularly in the field of medicine. However, the capacity of CRISPR-Cas9 to bind and cleave at locations other than the target site (termed off-targets), means great care must be taken when using it [4–7]. For this reason, many computational tools have been developed that seek to identify and predict potential off-targets and help inform experimental design [8–10].

Computational detection of off-targets consists of two components: identification and activity prediction. Identification involves identifying alternate sites the CRISPR-Cas9:gRNA complex may bind based on sequence complementarity. In addition to sequence similarity, potential off-targets must also be flanked by a Protospacer Adjacent Motif (PAM), a short sequence that the CRISPR-Cas complex must bind to in order to effect cleavage [11]. Detection of potential targets is usually achieved using traditional aligners such as BWA and Bowtie [8]. The likelihood that these target sites could become active off-targets can then be assessed using predictive models. While a number of scoring algorithms are available, the most common models for off-target activity prediction are the Cutting Frequency Determination (CFD) [12], the MIT score for off-target activity [13] and the recently developed Elevation score [9].

Bringing the search and scoring functionality together are pipelines such as CRISPOR [8], which uses BWA to identify potential off-targets and then evaluates them using either the CFD or MIT scores, as well as the Elevation pipeline [9], which uses a custom search tool and model of activity. These pipelines however do have limitations. While read-alignment-based tools offer fast off-target search, they are limited to very few mismatches between the gRNA and the off-target (typically 5). This is a substantial limitation as highly mutated off-targets with up to 8 mismatches have been recorded in experimental data [7, 14]. Additionally, current pipelines are not able to handle variant information. The genetic variations (SNPs, INDELs) found within an individual genome will change the off-target landscape [15–17]. This was recently demonstrated by Lessard et al., who showed experimentally that small variations in an off-target sequence could dramatically alter the cleavage rate of any given site [18]. It is therefore critical that the variant landscape of a genome be taken into account when designing CRISPR-Cas9 gRNAs, particularly for more personalized applications such as gene-therapy [16] and gene-drives [19].

To address both needs we developed VARSCOT (VARiant-aware detection and SCoring of Off-Targets). VARSCOT is able to process variant information provided as a VCF file to identify off-targets that are personalized to an individual. Furthermore, VARSCOT uses a novel seed-and-extend method [20] to allow more mismatches than other alignment-based tools (with a default of 8). VARSCOT also offers a novel machine-learning approach to score off-target activity by taking the sequence composition as well as the relationship between on- and off-targets into account.

Demonstrating the capabilities of VARSCOT, we firstly show how the target-site landscape dramatically changes when taking variant information into account. We then identify features that govern off-target activity and conclude by benchmarking VARSCOT against other activity-predictors as well as the state-of-the-art search-and-scoring pipelines.

### Implementation

#### Construction of the variant genome

VARSCOT integrates sequence variants of an individual from a user-defined VCF file by constructing a so-called variant genome that is scanned in addition to the reference genome. The variant genome sequences consist of 22 bp flanking regions upstream and downstream of a given variant that are extracted from the reference genome. For each allele, the corresponding variant is inserted into the sequence. Closely located variants that could potentially be included in a single off-target are extracted and further evaluated within a single sequence. Otherwise off-targets could be included that cannot exist if reference bases are extracted where an individual variant is located.

VARSCOT is intended to be used with phased variants since knowledge of the haplotypes is required in order to extract sequences with multiple variants correctly for each allele. In order to provide a method to process unphased variants, every possible combination of variants for each allele is reported within a sequence.

#### Read mapping based on Optimum search schemes

Targets are mapped to the reference and variant genome using a read aligner based on a bidirectional FM index. As opposed to traditional unidirectional indices, a bidirectional index can search into both directions in any given order thus improving runtime [21]. Most index-based approximate string matching strategies are still not practical for a large number of errors and already exceed acceptable running times for more than two errors. To allow for up to 8 errors in an index-based search, we use Optimum Search Schemes [20]. This is a strategy that enumerates a pattern with errors in a bidirectional index in such a way, that the number of steps in the index is reduced to a minimum. Using a recent implementation of the bidirectional FM-index based on EPR-dictionaries [22], which is faster by a factor of 2 for DNA alphabets than standard implementations of FM-indices based on Wavelet trees, we were able to reduce the search time even further.

After mapping the on-targets back to the reference and variant genome, both results are merged and filtered for the final output. Matches to the reference genome that lie within regions of individual variants need to be filtered out because they do not exist in the present individual and are covered by matches to the variant genome in the same regions. In addition, the original target sites are filtered out since they are always found as perfect matches by the aligner.

For all valid off-targets either the MIT score or Random Forest prediction can be calculated. The resulting off-targets and corresponding scores as well as their positional information and sequence are reported in an output file where the first columns correspond to a BED6 file. Each off-target that contains a variant is tagged as such with a reference back to the input VCF file.

#### Dataset curation

We employ two datasets in this study; a Training Dataset (9 on-target and 384 off-targets from [7]) and a Test Dataset (8 on-target and 5314 off-targets from [14]). In these studies, active off-targets were detected using the GUIDE-Seq or SITE-seq methods respectively. For the Training Dataset, we defined active off-targets as any that were detected using the GUIDE-Seq method. We therefore assumed that any sequence with up to 8 mismatches to the on-targets that were not detected were inactive off-targets. Because the number of inactive off-targets was larger than the active class, we performed down sampling. The sampling was weighted based on the mismatch distribution observed in the active off-targets to avoid any imbalances, as there are significantly more inactive off-targets with > 5 mismatches than in the active class. We repeated the sampling a total of 10 times, creating 10 sets of off-targets to avoid any sampling bias. For the Test Dataset, off-target activity was measured using different concentrations of CRISPR-Cas9. We defined off-targets as active if they were detected at a CRISPR-Cas9 concentration of 64 nM, the “standard” concentration used in the original paper.

#### Model training and feature selection

A Random Forest classifier was trained using 443 features derived from mismatch properties, sequence context and on-target activity (Additional file 2: Table S1) for each of the active-inactive off-target Training Dataset combinations. Feature importance was extracted for each model and then averaged across all repeats. Afterwards we performed feature selection using a backwards-selection method, where the least important feature is removed and the new model tested. Performance was measured using the out-of-bag error of the model and the combination of features which gave the lowest error were selected as the final model.

#### Predictive models

The standalone programs including off-target search for Elevation and CRISPOR were downloaded from their respective repositories. The CFD score was implemented using the scripts from [8] and the MIT off-target score was implemented in a python script using the weights provided in the original paper [13].

VARSCOT and Elevation were run on a 64-bit Linux system with 64 cores and 512 GB RAM. CRISPOR was run on a Macbook Pro with OS X 10.11, two cores and 16 GB RAM.

## Results

### VARSCOT identifies unique off-targets using variant information

To test VARSCOT’s ability to predict unique off-targets, we used VARSCOT to compare the predicted off-targets of 100 gRNAs across three individuals of the 1000 genomes project [23]. For this, we limited the prediction of off-targets to sites with up to five mismatches (the maximum number allowed by current state-of-the-art tools) and either the canonical NGG or non-canonical NGA PAM (the most active non-canonical PAM [24]). While the non-canonical NGA PAMA was chosen because it was found to be the most common alternative in experimental datasets [7], VARSCOT also allows users to specify additional non-canonical PAM’s to include in the off-target search.

**Fig. 1:**
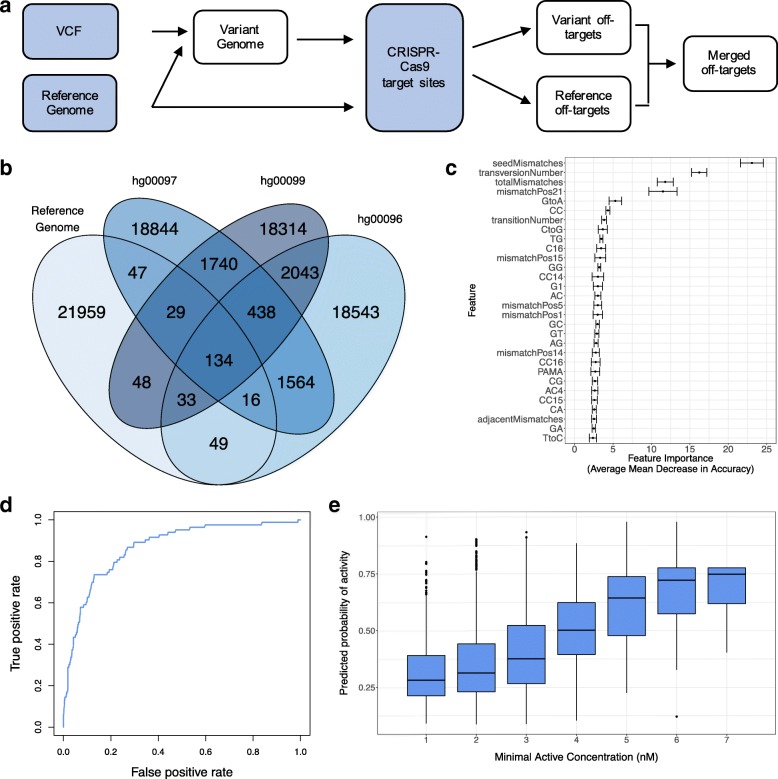
Development and testing of the VARSCOT model (**a**) VARSCOT uses a supplied VCF to produce a variant genome which is searched alongside the reference genome to identify variant off-targets. User supplied files are shown in blue, while files generated by VARSCOT are shown in white. **b** VARSCOT was used to detect potential off-targets for 100 gRNAs using variant information from three individuals from the 1000 Genomes project. Potential off-targets were compared between individuals and with the hg19 Reference Genome to identify unique targets. **c** Feature importance for predicting off-target activity. **d** Receiver Operating Characteristic (ROC) curve of the VARSCOT model tested on the independent Test Dataset filtered for targets with 8 or fewer mis-matches and NGG or NGA PAMs (**e**) Correlation of the VARSCOT Predicted Probability of Activity with the Minimal Active Concentration of CRISPR-Cas9 used in the Test Dataset

VARSCOT uses a supplied VCF file to generate a “variant genome” which, along with the reference genome, is searched using a seed-and-extend method based on Optimum Search Schemes using bidirectional FM indices [20] for regions similar to a supplied target sequence (Fig. 1a, a more detailed workflow is provided in Additional file 1: Figure S1). This search method allows VARSCOT to identify similar regions with up to 8 mismatches in a 23 bp sequence. Once identified, regions from the variant genome are compared to the reference genome to identify potential off-targets unique to the individual.

As shown in Fig. 1b, only 0.6% off-targets (134 sites) are consistent between all individuals and the human reference genome (hg19 assembly). In fact, the mutations an individual carries causes on average 98.97% of the off-targets (22,570 sites per genome, SE = 30) to be different when compared to the reference genome. Strikingly, the difference among the individuals is less with 81.68% of off-targets (18,626 sites per genome, SE = 125) unique to an individual. These results showcase the limitations of using a reference genome to identify off-targets for an individual and highlight the importance of understanding an individual’s variant landscape.

### The relationship between on- and off-target governs activity

Variants have the potential to create a significant number of new CRISPR-Cas9 binding sites, however binding does not always translate to cleavage. Similar to on-target activity [25], off-target activity can be predicted based on the sequence of the gRNA and the off-target [8, 9].

We trained a Random Forest classifier on a dataset where off-target activity was measured using the GUIDE-Seq method [7] (the Training Dataset) to take the single and di-nucleotide composition of a site as well as the number, position and type of mismatches into account when predicting off-target activity. Here, we chose to focus on off-targets with canonical PAM sequences only, as other PAMs were not well represented in the training set. We also limited the dataset to off-targets with only up to 8 mismatches. While off-targets with more mismatches have been reported, these are typically in ex situ experiments where the genomic DNA has been isolated and treated directly with CRISPR-Cas9 increasing the activity of even heavily mutated sites. In the Training Set, which is an in situ dataset, sites with more than 5 mismatches have a very low activity level hence likely rendering sites with more than 8 mismatches completely inactive (Additional file 1: Figure S2).

As the dataset only contains active off-targets, we assembled a list of inactive sites by randomly sampling the genome, matching the active off-targets in sequence complementarity and number of sites. To avoid selection bias, we repeat the sampling 10 times. For more details, see the methods section.

Using these datasets, we constructed 10 different models of off-target activity (one for each combination of active and inactive targets) and extracted the average feature importance across all models. Consistent with previous reports and as shown in Fig. 1c, features such as the number and position of mismatches were deemed important, particularly if the mismatches fell within the seed-region (the 12 bp immediately upstream of the PAM). In addition, it was also found to be important whether the mismatch was caused by a purine/pyrimidine (transversion) or purine/purine or pyrimidine/pyrimidine substitution (transition), suggesting that structural differences between the gRNA and potential off-target influence overall activity.

Interestingly, whether the first base of the PAM at the off-target position matched that at the on-target position, was the 57th most important feature in our model. This position is known to be important for regulating on-target activity of a gRNA [25]. We hence hypothesize that the model uses it to estimate on-target activity as an influencing factor on off-target activity.

In order to identify the minimal number of features required to accurately model off-target activity, we performed feature selection using a backwards-selection strategy, identifying 80 key features. On average a cross-validated Area Under the Curve (AUC) of 0.956 (SE = 0.005) was achieved for training with selected features only and 0.955 (SE = 0.006) for training with all features (Additional file 1: Figure S3). This suggests that feature selection does not significantly improve performance (paired *t*-test *p* = 0.41) but rather allows us to exclude unnecessary information. From the 10 training sets, we selected the best-performing model as final model for validation.

### Off-target activity can be modelled using only the target sequence

To confirm the model generalizes after feature-selection and training we validate its performance on an independent Test Dataset by Cameron et al. [14]. This Test Dataset consists of off-targets that were detected across a range of CRISPR-Cas9 concentrations. For this validation test, we considered an off-target active if it was active at a CRISPR-Cas9 concentration of 64 nM (the standard concentration used by Cameron et al.). Testing our model on this dataset yields an AUC of 0.85 (Fig. 1d). This is especially remarkable as our model was trained on off-targets with canonical PAMs (AUC of 0.86 for canonical and 0.83 for non-canonical PAMs, Additional file 1: Figure S4). This indicates that off-target activity is primarily driven by the target sequence of the off-target and the gRNA and not the PAM.

We also tested if the predicted activity score correlates with the concentration-dependent activity of the off-targets in the Test Dataset. We divided off-target sites in the Test Dataset into groups based on the minimum CRISPR-Cas9 concentration they were active at (with a lower minimum concentration equalling a more active off-target) and plotted the corresponding average predicted activity score from our model. Our results show a clear correlation between activity-score and concentration-score (Fig. 1e), suggesting that our model can also be used to predict activity of off-targets at different CRISPR-Cas9 concentrations.

### Comparison with other scores for off-target activity

We compared our model with the previously published off-target activity scorers, the MIT [13] and CFD score [12] as well as the Elevation score [9]. These were shown to outperform other available scores in a recent review and therefore represent the currently best scoring schemes [8].

**Fig. 2:**
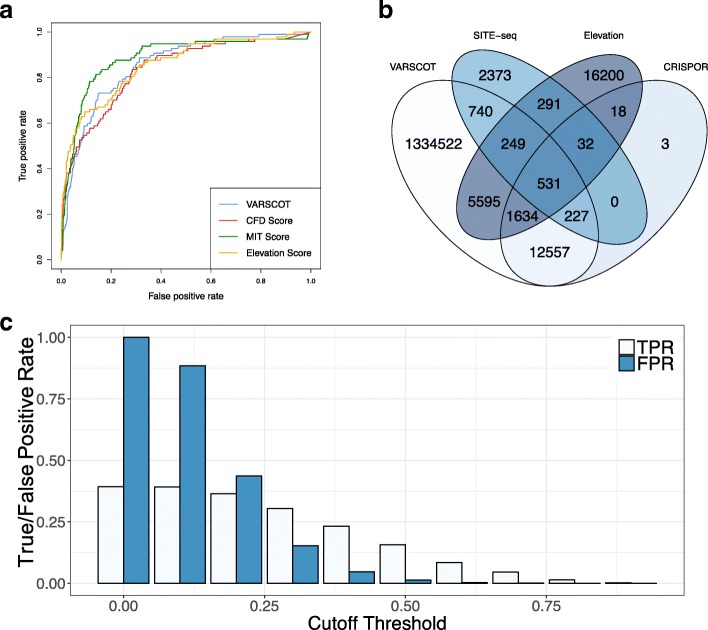
Comparison of the VARSCOT pipeline (**a**) Receiver Operating Characteristic (ROC) curves of VARSCOT and other off-target activity models tested on the Test Dataset. **b** Number of potential off-targets detected by the VARSCOT, Elevation and CRISPOR pipelines compared to the off-targets detected experimentally in the Test Dataset. **c** Effect of a threshold on the True Positive Rate (TPR) and False Positive Rate (FRP) of detection of VARSCOT

Figure 2a shows the resulting ROC curves on the independent Test Dataset. All models showed strong performance with AUCs > 0.83. Pairwise comparison showed that only the MIT and Elevation as well as the MIT and CFD scores were significantly different, with the MIT score outperforming both (*p*-values = 0.009 for both comparisons, all other comparisons *p*-value > 0.05). Because there was no significant difference between the performance of our and the MIT model, we elected to include both in the final VARSCOT pipeline.

### The VARSCOT pipeline outperforms current off-target detection and activity prediction pipelines

The performance of VARSCOT was compared to the state-of-the-art off-target detection and activity prediction pipelines Elevation [9] and CRISPOR [8]. VARSCOT was run allowing up to 8 mismatches, while CRISPOR was limited to 5 mismatches and Elevation allowed 6 (up to 3 within the 17 bases proximal to the PAM and any number of mismatches in the three most distal bases) mismatches, respectively. For CRISPOR this was due to the limitations in BWA and for Elevation this was due to runtime as a search with comparable mismatch-number would have taken an order of magnitude longer (hours for a single on-target compared to minutes for both other methods). In order to enable a fair comparison, VARSCOT was used without variant information and Elevation and VARSCOT were limited to canonical NGG and non-canonical NGA PAMs.

As shown in Fig. 2b, of the 4443 sites in the Test Dataset VARSCOT identifies the most out of all methods (1747, 39% of observed sites), followed by Elevation (1103, 25%) and CRISPOR (790, 18%). Of the missed sites, 77% (2078 sites) were missed due to VARSCOT limiting detection to off-targets with up to 8-mismatches, and the remaining 23% (618 sites) were missed due to the presence of non-canonical PAMs. Critically, the missed sites were predominantly low-activity off-targets confirming that VARSCOT identifies the active off-targets of interest (Additional file 1: Figure S5).

All three pipelines report more off-targets than are reported in the Test Dataset. VARSCOT identifies an additional 1,354,308 sites, while CRISPOR and Elevation report an additional 14,212 and 23,447 sites respectively. In order to filter out false-positives, a probability cut-off based on our predicted score can be used. Using a cut-off threshold of 0.5 reduces the number of false positives from 1,356,055 to 18,764 (a reduction of approximately 98%). While a higher threshold will reduce this further, it also reduces the number of true positives. Care must therefore be taken when choosing a threshold, although it is critical to note that the false positives reduces at a faster rate than the true positives (Fig. 2c). Using a cut-off of 0.4 yields a true-positive rate of 23% and a false-positive rate of 5%.

## Discussion

VARSCOT is a newly developed off-target detection and scoring tool for CRISPR-Cas9, which incorporates the variant information of individuals into the search. We have shown that when considering the specific genetic landscape of an individual, 99% of off-targets are unique and would be missed when scanning a reference genome only. Hence SNP-aware off-target detection is critical for any application of CRISPR which requires an element of personalization, such as gene-therapy [16]. VARSCOT is also capable of handling population level variant information. This will be of great use in fields such as gene-drives [19], where individual genome variants about the targeted species cannot be known but population level information on genetic variation at specific loci is available.

VARSCOT detects off-targets in the variant and reference genome using a method based on Optimum Search Schemes using a bidirectional FM index, which is more sensitive and identifies off-targets with more mismatches than traditional aligners. While the default of allowing up to 8 mismatches means VARSCOT captures more validated off-targets than other pipelines, this cutoff can be increased to identify more divergent off-targets. However, this would increase the possibility of false positives which must then be accounted for.

The effect of false positives can be mitigated by using a model to predict the activity of a potential off-target such as the one we developed. While we limited the Training data of our model to only off-targets with NGG or NGA PAMs, critically our model could accurately predict the activity of off-targets with other PAMs (Additional file 1: Figure S4b). In the Test Dataset, applying a standard cut-off of 0.5 reduced the number of false positives by approximately 98%. Deciding on a probability threshold will be a critical step for future experimental design and the correct threshold will depend on the parameters.

Our results showed that the predicted on-target activity of a gRNA is an important factor of off-target activity, suggesting that more care should be taken with gRNAs selected for on-target activity as they will likely have more active off-targets. Similarly, an experiment that uses a higher concentration of CRISPR-Cas9 should be cautious, as previously inactive off-targets could become active.

## Conclusions

Natural genomic variants can have a profound impact on the off-target activity of CRISPR-Cas9 and accounting for this variation is therefore critical. VARSCOT is the first off-target detection tool that can account for genetic variation and identify off-targets unique to an individual genome. This will be critical for future work seeking to apply CRISPR-Cas9 to wild type populations or potentially in the clinic.

### Availability and requirements

Project name: VARSCOT.

Project home page: https://github.com/BauerLab/VARSCOT

Operating system(s): Platform independent.

Programming language: Python and C++.

Other requirements: Python 2.7 with numpy, scipy, sklearn 0.19.0 and pybedtools. R3.4.2 with randomForest. CMake and gcc.

License: CSIRO Non Commercial Source Code License Agreement v1.0.

Any restrictions to use by non-academics: License required for non-academic use.

## Additional files

Additional file 1: **Figure S1.** Overview of the VARSCOT pipeline. **Figure S2.** Correlation of activity and mis-match number. **Figure S3.** Results of Feature Selection. **Figure S4.** Performance of the VARSCOT model. **Figure S5.** Distribution of detected and non-detected off-targets. (DOCX 440 kb)

Additional file 2: **Table S1.** Feature list for predictive model of off-target activity. (DOCX 16 kb)

## Data Availability

All data analyzed here as well as the VARSCOT pipeline are available from https://github.com/BauerLab/VARSCOT
