# Supplementary material for: VARSCOT: variant-aware detection and scoring enables sensitive and personalized off-target detection for CRISPR-Cas9
Source: BMC Biotechnol. 2019 Jun 27;19:40. doi: 10.1186/s12896-019-0535-5 (PMC6598273; doi:10.1186/s12896-019-0535-5)
Supplement: Supplementary file 1 — Figure S1. Overview of the VARSCOT pipeline. Figure S2. Correlation of activity and mis-match number. Figure S3. Results of Feature Selection. Figure S4. Performance of the VARSCOT model. Figure S5. Distribution of detected and non-detected off-targets. (DOCX 440 kb) [file 12896_2019_535_MOESM1_ESM.docx]

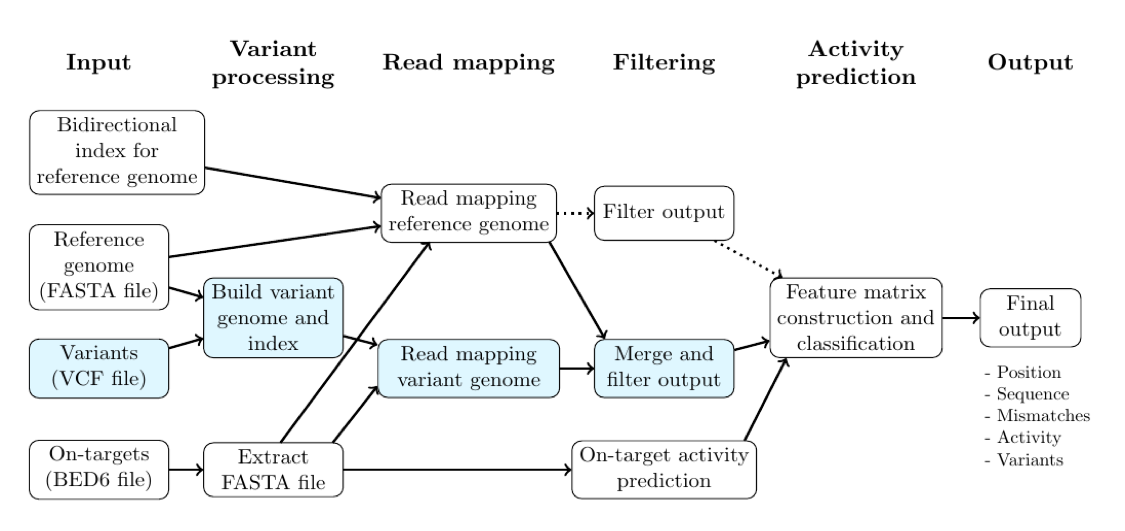


**Additional file 1: Figure S1. Overview of the VARSCOT pipeline.** As a minimum input VARSCOT takes a reference genome and the corresponding bidirectional FAMOUS index. A variant genome is constructed from the supplied VCF file and scanned in addition to the reference genome for off-targets. Results from both mappings are merged and filtered and finally off-target candidates are scored using either the VARSCOT model or the MIT off-target score.


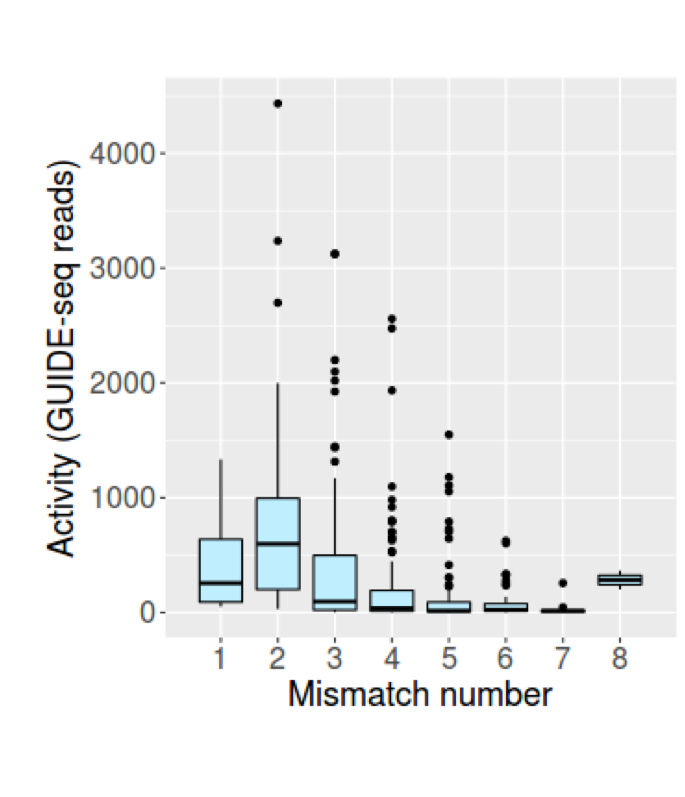


**Additional file 1: Figure S2. Correlation of activity and mis-match number.** Activity of potential off-targets from the Training Dataset (measured by the number of GUIDE-seq reads) was compared to the number of mis-matches between the off-target and the gRNA.


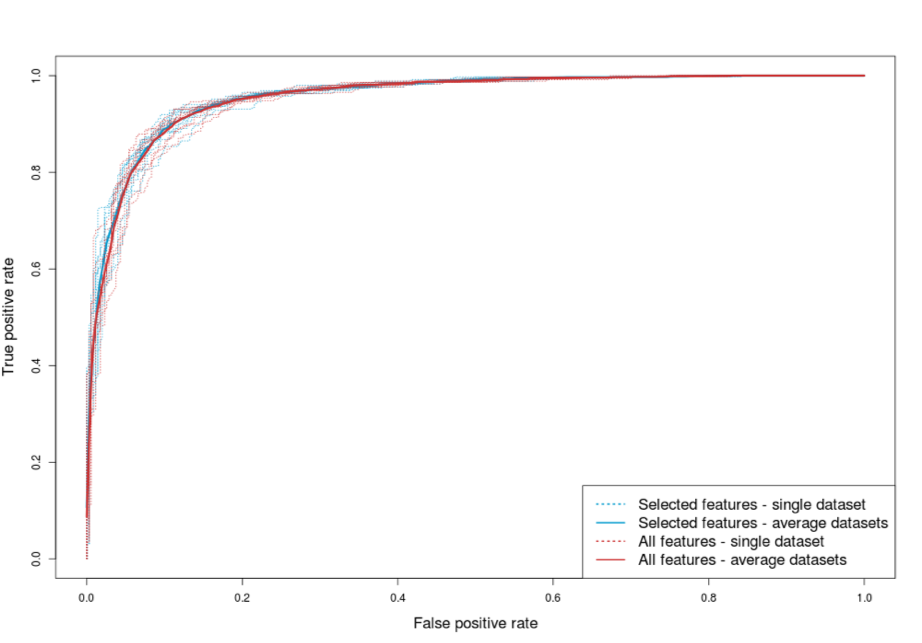


**Additional file 1: Figure S3. Results of Feature Selection.** Training performance of the Random Forest with and without feature selection on all ten datasets. All datasets perform well with an average AUC of 0.956 for training with selected features and 0.955 for all features.


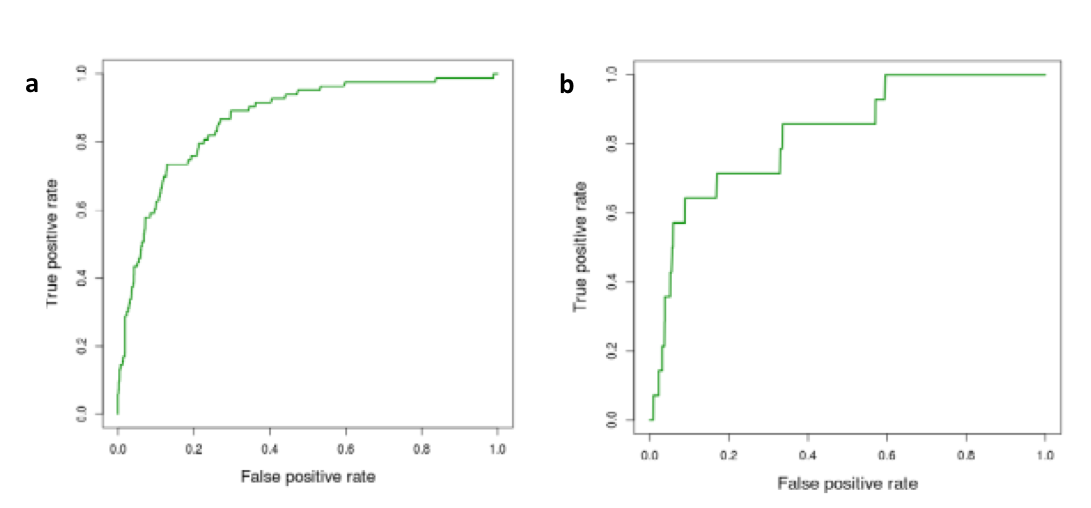


**Additional file 1: Figure S4. Performance of the VARSCOT model.** Receiver Operating Characteristic (ROC) curve of the VARSCOT model tested on the independent Test Dataset filtered for targets with 8 or fewer mis-matches and NGG or NGA PAMs (a) or all other PAMs (b)


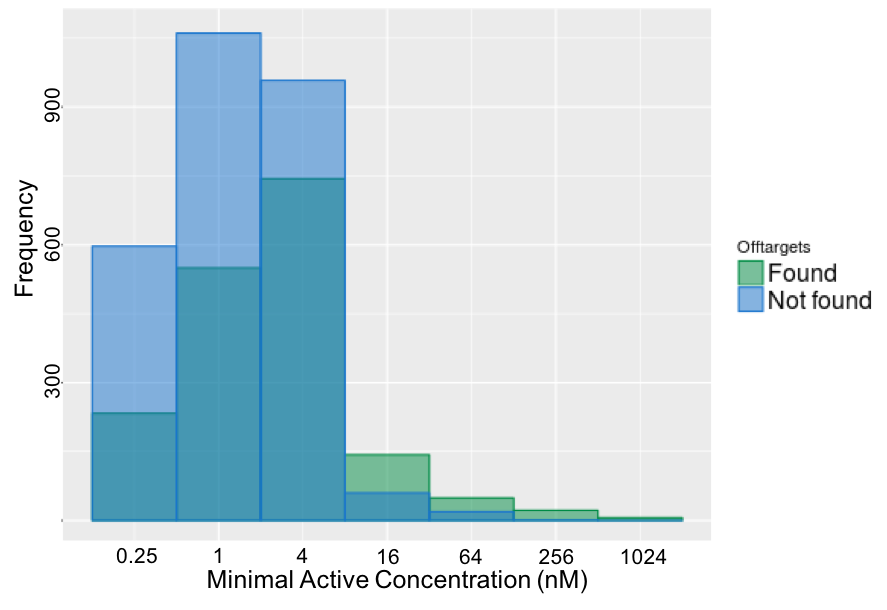


**Additional file 1: Figure S5. Distribution of detected and non-detected off-targets.** Comparison of the minimal active Cas9 concentration of the off-targets detected by the VARSCOT pipeline (green) and those that were not detected (blue) from the Test dataset.
