# Supplementary material for: VARSCOT: variant-aware detection and scoring enables sensitive and personalized off-target detection for CRISPR-Cas9
Source: BMC Biotechnol. 2019 Jun 27;19:40. doi: 10.1186/s12896-019-0535-5 (PMC6598273; doi:10.1186/s12896-019-0535-5)
Supplement: Supplementary file 2 — Table S1. Feature list for predictive model of off-target activity. (DOCX 16 kb) [file 12896_2019_535_MOESM2_ESM.docx]

| **Feature Group** | **Explanation** | **Number** | **Type** |
| --- | --- | --- | --- |
| Single letters per position | Single letter at each position except PAM (A, C, G or T at position 1 to 20) | 80 | Binary |
| Paired letters per position | Paired letters at each position except PAM (AA, AC, …, TT at position 1 – 19) | 304 | Binary |
| Overall pair count | Total number of paired letters except PAM | 16 | Numeric |
| Mismatch positions | Mismatch in comparison to on-target at position 1 to 21 | 21 | Binary |
| Type of mismatch | Type of mismatch at any position (e.g. A to G) | 12 | Binary |
| Transversion number | Number of changes from purine to pyrmidine (A or G to C or T and vice versa) | 1 | Numeric |
| Transition Number | Number of changes from purine to purine or pyrmidine to pyrmidine (A to G or C to T and vice versa) | 1 | Numeric |
| Number of mismatches | All mismatches in comparison to on-target | 1 | Numeric |
| Number of mismatches in seed region | Mismatches within 12 bases proximal to PAM | 1 | Numeric |
| Number of adjacent mismatches | Mismatches that lie adjacent to another mismatch | 1 | Numeric |
| Activity of corresponding on-target | Predicted by TUSCAN | 1 | Numeric |
| PAM letter | The letter at N-position of the NGG PAM | 4 | Binary |

Table S1: Feature list for predictive model of off-target activity
